# The Mutant Form of Lamin A that Causes Hutchinson-Gilford Progeria Is a Biomarker of Cellular Aging in Human Skin

**DOI:** 10.1371/journal.pone.0001269

**Published:** 2007-12-05

**Authors:** Dayle McClintock, Desiree Ratner, Meepa Lokuge, David M. Owens, Leslie B. Gordon, Francis S. Collins, Karima Djabali

**Affiliations:** 1 Department of Dermatology, College of Physicians and Surgeons, Columbia University, New York, New York, United States of America; 2 Department of Pathology, College of Physicians and Surgeons, Columbia University, New York, New York, United States of America; 3 Department of BioMed Pediatrics, Brown University, Providence, Rhode Island, United States of America; 4 Genome Technology Branch, National Human Genome Research Institute, National Institutes of Health, Bethesda, Maryland, United States of America; University of Florida, United States of America

## Abstract

Hutchinson-Gilford progeria syndrome (HGPS, OMIM 176670) is a rare disorder characterized by accelerated aging and early death, frequently from stroke or coronary artery disease. 90% of HGPS cases carry the *LMNA* G608G (GGC>GGT) mutation within exon 11 of *LMNA,* activating a splice donor site that results in production of a dominant negative form of lamin A protein, denoted progerin. Screening 150 skin biopsies from unaffected individuals (newborn to 97 years) showed that a similar splicing event occurs *in vivo* at a low level in the skin at all ages. While progerin mRNA remains low, the protein accumulates in the skin with age in a subset of dermal fibroblasts and in a few terminally differentiated keratinocytes. Progerin-positive fibroblasts localize near the basement membrane and in the papillary dermis of young adult skin; however, their numbers increase and their distribution reaches the deep reticular dermis in elderly skin. Our findings demonstrate that progerin expression is a biomarker of normal cellular aging and may potentially be linked to terminal differentiation and senescence in elderly individuals.

## Introduction

Lamins of the A- and B-type are intermediate filament proteins that constitute major components of the nuclear lamina, a filamentous meshwork forming an interface between the inner nuclear membrane and the chromatin [1]. Lamins A and C, the major isoforms of A-type lamins, are expressed in all differentiated vertebrate cells [2] and are translated from alternatively spliced transcripts of the *LMNA* gene. In contrast to the single *LMNA* gene, there are two B-type lamin genes: *LMNB1* gene encodes lamin B1 protein [3], [4], while *LMNB2* encodes two protein products by alternative splicing: lamin B2 and lamin B3, [5], [6]. The B-type lamins are expressed throughout development, and one or more B-type lamins are present in all cell types [7]–[9]

Lamins are located at the nuclear lamina and throughout the nucleoplasm [10], [11], where they seem to play fundamental roles in the shape, integrity and function of the nucleus and in DNA replication and RNA transcription [12]. Lamin A and lamin B are modified at their carboxyl-terminal –CAAX box through a series of post-translational modifications. The modifications include, successively, farnesylation of the cysteine in the C-terminal CaaX motif (C, cysteine; a, aliphatic; X, any amino acid), followed by a proteolytic cleavage of the aaX-terminal tripeptide, and by methylation of the farnesylated cysteine [13]. While B-type lamins remain permanently farnesylated, prelamin A (the precursor of mature lamin A) undergoes a second cleavage of the remaining 15 C-terminal residues (aa 647–661) to give rise to the mature lamin A, therefore losing its farnesyl modification [13], [14]. The enzyme responsible for these sequential proteolytic cleavages is the zinc metalloproteinase ZMSPTE24, for which lamin A is the only known substrate in mammals [15].

Mutations in *LMNA* are implicated in 12 distinct disorders, commonly referred to as laminopathies, and involve different tissues, including muscle, peripheral nerve, adipose, bone and skin tissue. These disorders exhibit distinct clinical phenotypes associated with features such as myopathy, cardiomyopathy, lipodystrophy, neuropathy and premature aging [16]–[18]. The two best-known examples of accelerated aging syndrome in humans are Hutchinson-Gilford progeria syndrome (HGPS, ‘Progeria of childhood’) and Werner syndrome (WS, ‘Progeria of the adult’). Whereas most cases of WS have been caused by mutations in WRN helicase [19], a subset of WS patients do not show mutations at the WRN locus (atypical WS), but show heterozygous amino acid substitutions in the heptad repeat region of lamin A [20]–[22].

Hutchinson Gilford progeria syndrome (HGPS, OMIM 176670) is a rare sporadic disorder with an incidence of 1 per 4–8 million live births, consisting of a premature aging phenotype with rapid growth deceleration in childhood [18]. Appearance at birth and birth weight are usually normal, but growth is typically slowed by the age of one year [23]. The phenotypic appearance consists of the following: short stature, sculpted nose, alopecia, prominent scalp veins, loss of subcutaneous fat, and dystrophic nails. In addition, HGPS patients show skeletal abnormalities that may reflect deficient osteogenesis, principally in the extremities, mandibular and cranial dysplasia with disorganized growth, deformations in dentition and severe osteolysis [24] [25]. The common causes of death in HGPS subjects during the second decade of life are chronic conditions most common in elderly people, especially coronary artery disease and stroke due to widespread arteriosclerosis [26].

Nearly 90% of the subjects affected with HGPS carry a *de novo* G608G (GGC>GGT) mutation within exon 11 of *LMNA,* [22], [27], [28]. This single nucleotide change activates a cryptic splice donor site, which results in a deletion of the 3′ terminal 150 nucleotides of exon 11 of the mRNA, causing a 50 amino acid internal truncation near the carboxyl-terminus of prelamin A [28]. The truncated lamin A, referred to as progerin, lacks amino acids 607 to 656 of prelamin A but retains the CAAX box [22], [27], [28]. Because the endoproteolytic cleavage site for ZMPSTE24 is lost in the mutant protein, progerin remains permanently farnesylated causing its tight association with the nuclear envelope and producing numerous nuclear envelope abnormalities [29], [30]. This modification also appears to affect the dynamic state of progerin within the lamina [31], [32].

Cells derived from HGPS individuals and subjects with pathologies resembling HGPS, such as atypical progeria Werner Syndrome (WS), Restrictive Dermopathy (RD), and Mandibular Dysplasia (MAD), appear to share a common feature: accumulation of prelamin A, either as full-length prelamin A protein or various truncated forms of prelamin A [14]. In all of these diseases, the prelamin A or mutant prelamin A remains farnesylated and accumulates within the nuclear compartment as cellular generation increases [29], [30], [33]. The persistence of the farnesylated form appears to be the key element responsible for severe nuclear abnormalities and defects in heterochromatin organization, mitosis, DNA replication, transcription and repair [34]–[37].

Recently, rare fibroblasts cultured from elderly individuals were found to exhibit nuclear phenotypes identical to those of HGPS cells [38]. Intriguingly, while those cells expressed progerin mRNA transcripts at barely detectable levels [38], long-term cultures contained a few abnormal nuclei that were clearly positive with anti-progerin specific antibody [39]. These observations indicate that progerin is also expressed in normal cells. Presumably, the cryptic splice donor site in exon 11 of LMNA is activated by the HGPS mutation, but the normal sequence is able to function in a similar fashion under some circumstances, at least in long-term culture.

To further address the biological relevance of progerin expression in unaffected individuals and its relationship to normal aging, we followed the spatiotemporal expression pattern of progerin in human skin at all ages. Herein, we provide new evidence indicating that progerin is expressed and accumulates *in vivo* during normal aging.

## Results

### In vivo detection of progerin in unaffected individuals

**Figure 1:**
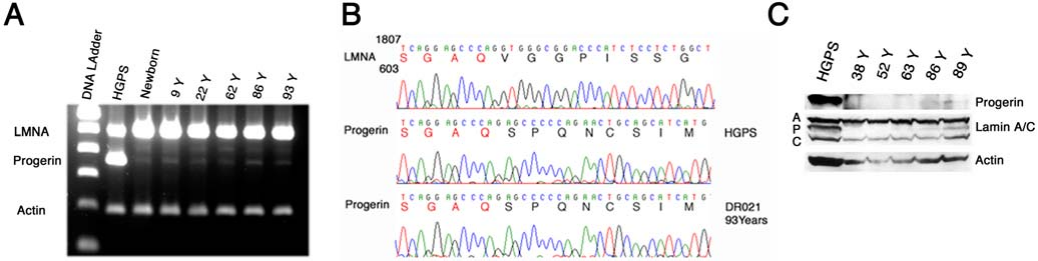
Progerin expression in human skin. A, RT-PCR analysis of HGPS cells and human skin biopsies of indicated age, primers amplifying wild-type and progerin transcripts. B, Direct sequencing of a short portion within exon 11 of wild type lamin A (LMNA), and progerin transcripts from HGPS and 93-year-old subjects. C, Western blot analysis of protein extracted from skin biopsies of indicated age with anti-progerin 972S9, anti-lamin A/C and anti-actin antibodies.

Using the human skin as our model system, we investigated whether progerin is expressed in the skin of unaffected individuals. 150 skin biopsies from newborn foreskins and from unaffected individuals, including equal numbers of females and males ranging in age from 22 to 97 years were collected from the Dermatology Clinic at Columbia University. The biopsies originated from different body sites (Table 1). Using a one-step reverse transcription polymerase chain reaction (RT-PCR), we screened total RNA preparations isolated from skin biopsies. Primers in exon 9 and exon 12, described previously [38], primarily amplified wild type lamin A; however, a minor fragment similar in size to the HGPS transcript was detected in 50 biopsies, examples of which are shown in Figure 1A. Strikingly, the levels of amplified short product remained low in all samples and no age-related differences were observed. Sequencing of the short cDNA product derived from RNA preparation of a 93 year-old donor was found to be identical to the progerin cDNA sequence (Fig. 1B), demonstrating that the progerin transcript, previously identified as an aberrant lamin A product in HGPS, might constitute a true physiological lamin A isoform. Another minor cDNA fragment migrating slightly higher than the progerin product was detected in all skin samples and was identified as the delta 10 isoform of lamin A [40].

**Table 1:** Human skin biopsies

Age	Body site	Number of Subjects
Newborn	Foreskin	8 M
22 to 46	Breast	6 F
30 to 55	Face (cheek, forehead, scalp, ear, nose) Leg	8 F/10 M
56 to 70	Face (temple, cheek, forehead, scalp, ear, nose)	17 F/10 M
56 to 70	Neck, chest, Leg, back, hand	5 F/6 M
71 to 97	Face (temple, cheek, forehead, scalp, ear, nose)	26 F/33 M
71 to 97	Neck, chest, arm, Leg, back, hand	10 F/11M

Classification of the skin biopsies originating from different body sites of unaffected individuals and collected at the Dermatology Department in accordance with the Columbia University Board protocols on human subject protection.

Total# 150 Samples

The ubiquitous presence of a low level of progerin mRNA in human skin also suggested that the protein, if present, would be expressed at very low levels. We previously generated a rabbit polyclonal antibody specific to lamin A G608G (progerin) [30]. This previous polyclonal anti-progerin did not detect progerin in normal tissue, and to rule out the possibility of the antibody being of low affinity or that the recognized epitope might be masked, we generated a rabbit monoclonal antibody using the same peptide as antigen as described in Materials and Methods [30]. Three rabbits were immunized. The serum of rabbit 972 specifically recognized progerin protein and gave no signal with A-type lamin including pre-lamin A (data not shown). Spleen derived lymphocytes isolated from rabbit 972 were used to generate hybridomas (Epitomics, Inc.; Burlingame, CA, USA). One clone, 972S9, was selected based on its specific reaction for progerin and was used in this study. We screened 40 skin biopsies of different ages for progerin expression by Western blot analysis, and as indicated in a representative blot, a small amount of progerin protein was present in skin samples derived from elderly individuals (Fig. 1C). Progerin was not detected in protein extracts derived from young adult skin under our experimental conditions, but low levels of expression were clearly detected in samples derived from old skin and appeared to increase slightly with the increasing chronological age of the donor (Fig. 1C).

### Normal fibroblasts overexpressing progerin recapitulate HGPS cellular phenotype

**Figure 2:**
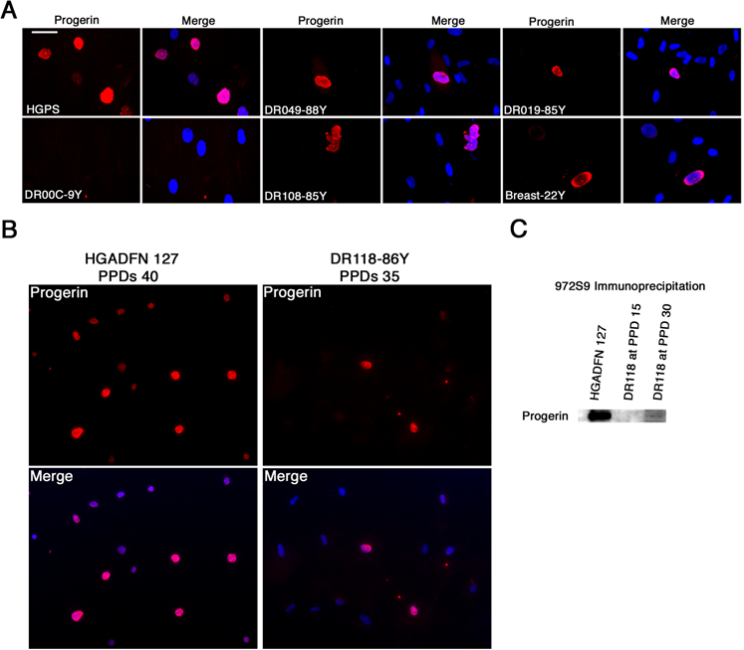
Progerin expression in primary dermal fibroblast cultures. A, Immunofluorescence microscopy on primary dermal fibroblasts from an HGPS subject and unaffected individuals of indicated ages with rabbit monoclonal anti-progerin antibody. B, Immunofluoresence detection of progerin in HGADFN 127 (HGPS individual) and DR118 (86-year-old individual) at late PPDs. C, Western blot analysis of nuclear protein extracts derived from fibroblast cultures HGADFN 127 (HGPS) at PPD 20 and DR118 (86-year-old female) at PPD 15 and 30, respectively, were immunoprecipitated with anti-progerin mAb 972S9 and the corresponding Western blot was probed with the same antibody.

We established primary fibroblast cultures from skin biopsies of normal individuals at different ages (Table 1). Primary fibroblast cultures were made using two different methods either by explant outgrowth or by enzymatic dissociation of the dermis [41]. Indirect immunofluorescence analyses were performed with the anti-progerin monoclonal antibody on fibroblast cultures at early population doublings (PPDs). HGPS cultures below 25 PPDs showed that 27% of nuclei were progerin-positive. In primary fibroblast cultures from unaffected individuals, a few nuclei showed a positive staining (Fig. 2A). Independently of the method used for their establishment, cultures derived from young subjects (22 to 30 years) showed less than 0.01% progerin-positive staining, while cultures derived from elderly subjects exhibited an average of 0.3% to 0.8% progerin-positive nuclei at early PPDs. Progerin-positive cells from normal individuals showed nuclear abnormalities similar to those observed in HGPS fibroblasts (Fig. 2A). Nuclear blebs, nuclear envelope invaginations, binucleated cells and large nuclei, reminiscent of abnormal cell cycle exit, were observed in all cultures derived from elderly individuals, as was recently reported [39].

When fibroblast cultures were serially passaged to approach the end of their lifespan, HGADFN 127 cells (HGPS) ceased to grow when they reached approximately 40 to 45 PPDs under our culture conditions. Indirect immunofluorescence analyses with anti-progerin rabbit mAb 972S9 of HGPS cultures showed an average of 27% of progerin-positive nuclei at early PPDs, while towards late PPDs (above 35) nearly 90% of the cells were progerin-positive (Fig 2 B). When similarly studies were performed with normal fibroblast cultures, we noticed that the number of cells positively labelled with anti-progerin Ab increased slightly with the cellular-age in vitro. As exemplified, fibroblast culture DR118 established from an 86 year-old female was monitored by indirect immunofluorescence with anti-progerin mAb and exhibited an average of 0.4% of progerin-positive cells in young cultures. That average increased to 0.8% in late cultures (PPDs 30 to 35). Western blot analysis of total protein extracts isolated from primary fibroblast cultures derived from adult and elderly individuals exhibited no progerin signal when probed with anti-progerin mAb, while progerin was readily detected in total HGPS cellular extracts at early PPDs (data not shown). Previously, we reported that progerin accumulates in HGPS fibroblasts in a cellular-age-dependent manner [30]. To verify further whether normal fibroblasts could accumulate progerin in a similar fashion to the HGPS counterpart, we performed immunoprecipitation assays with anti-progerin mAb using nuclear extract preparation. Immunoprecipitation of progerin was performed on isolated nuclei preparation from an average of 6×10^6^ of normal cells from elderly individuals at early and late PPDs (Fig. 2C). Western blot analyses of immunoprecipitated materials were probed again with anti-progerin mAb. As indicated in Figure 2C, a low amount of progerin could be detected at late PPDs in a representative fibroblast culture, DR118 that was established from an 86 year-old female (Fig. 2C).

Collectively, these results indicate that low levels of progerin protein are present in skin derived from elderly individuals as well as in primary dermal fibroblast cultures established from this population of subjects. Importantly, progerin appeared to accumulate in normal fibroblast cultures in a cellular-age-dependent manner, but remained still relatively low when compared to the amount of progerin accumulating in HGPS cultures [29], [30].

### *In vivo* profiling of progerin protein on skin biopsies derived from HGPS and unaffected individuals using an anti-progerin monoclonal antibody

**Figure 3:**
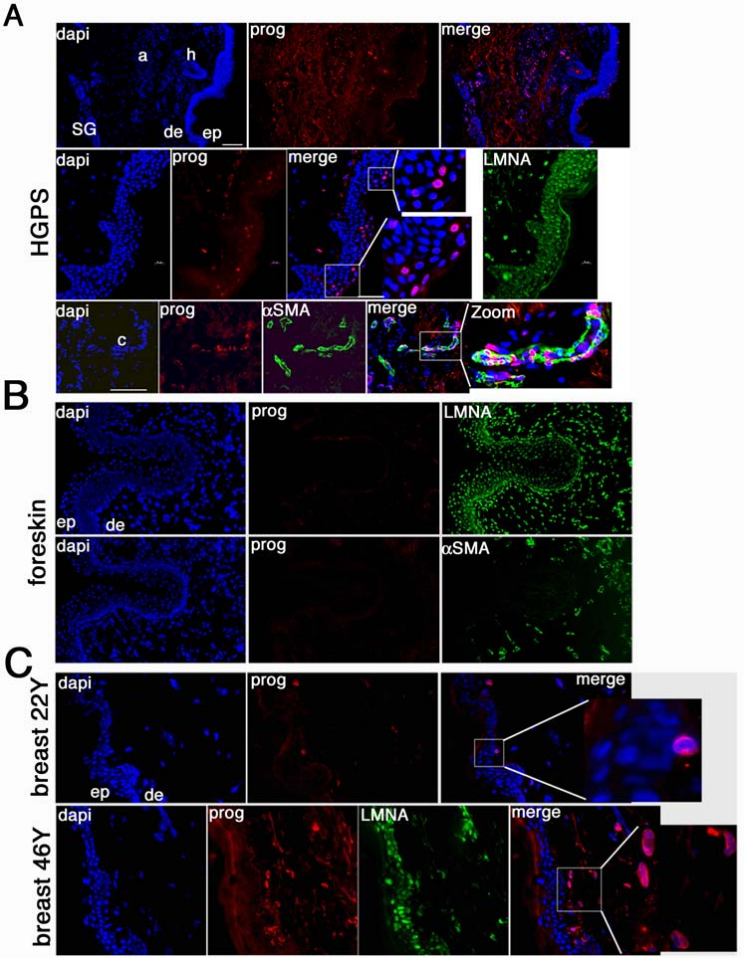
In situ localization of progerin on human skin sections derived from a subject with HGPS and from unaffected individuals. A, HGPS skin sections immunostained with anti-progerin (prog), anti-lamin A (LMNA) antibody, or anti-α smooth muscle actin antibody (αSMA) and counterstained with a DNA stain (dapi). Morphologic entities are indicated: epidermis (ep), dermis (de), sweat glands (SG), capillary (c), arrector pili muscle (a), and hair follicle (h). B, Newborn foreskin sections immunolabelled with anti-progerin or anti-αSMA antibody. C, Breast skin sections from 22- and 46-year-old female subjects probed with anti-progerin and lamin A. The respective double or triple merged signals are indicated.

To gain insight into the functional impact of the progerin isoform, we examined human skin biopsies to determine the cellular distribution of progerin *in vivo*. In skin sections derived from a 9 year-old subject with HGPS (HGADFN143), we previously demonstrated that progerin was localized in vascular cell nuclei and in nuclei throughout the dermis [30]. Using the monoclonal anti-progerin Ab, we reanalyzed sections from the same HGPS sample. Progerin was detected within dermal nuclei, blood vessels, arrector pili muscle, and cells surrounding the sweat glands (Fig. 3A). Furthermore, progerin was detected in keratinocyte nuclei in the upper most layers of the epidermis and localized into a thick rim like structure at the nuclear envelope. This distribution indicates that a subset of terminally differentiated keratinocytes accumulates progerin in HGPS (Fig. 3A).

**Figure 4 pone-0001269-g004:**
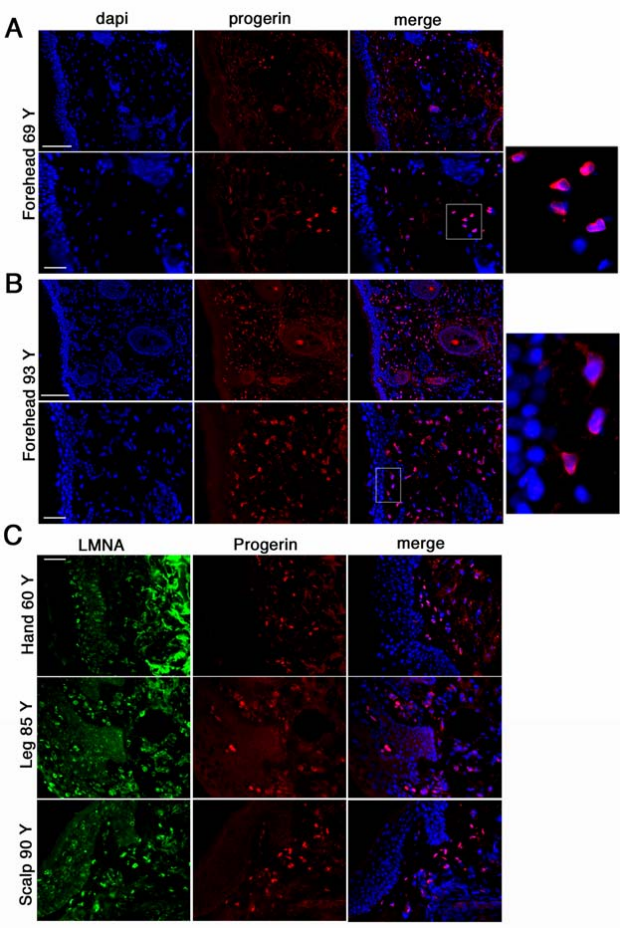
Progerin accumulation in human elderly skin biopsy sections. A, Forehead skin section from a 69-year-old individual probed with anti-progerin antibody and counterstained with dapi. Bars correspond to 100 and 50 µm, respectively. B, Forehead skin section from a 93-year-old donor. C, Skin sections from different body sites as indicated were probed with anti-progerin and anti- lamin A (LMNA) antibodies. Bar, 50 µm. Merged images are indicated.

Since low levels of progerin mRNA were detected in total skin mRNA preparations at all ages (Fig. 1A) and low amounts of protein were detected in elderly skin extracts (Fig. 1C), we hypothesized that progerin would be present at low levels or in only a few cells. We performed immunohistochemistry on sections derived from newborn foreskins and sixty skin biopsies from different body sites (Table 1) of 22 to 93-year-old subjects. Representative patterns of progerin localization are shown in Figures 3 and 4. Newborn foreskin exhibited no signal with anti-progerin mAb, while all nuclei showed a positive signal with anti-lamin A Ab (Fig. 3C, panel LMNA). We noted that the upper dermis on serial foreskin sections was highly vascularized; numerous vascular loops were labelled with anti-α smooth muscle actin Ab (αSMA) (Fig. 3B). Screening of five foreskin biopsies detected no progerin in any skin compartment. Breast skin sections from a 22-year-old female showed a few progerin-positive nuclei close to the basement membrane and in the papillary dermis (Fig. 3C). Breast skin sections from a 46-year-old woman exhibited a greater number of progerin-positive nuclei dispersed throughout the papillary dermis (Fig. 3C).

Sections from the forehead of a 69-year-old male, showed progerin-positive nuclei mostly in the upper dermis (Fig. 4A). Skin sections from the forehead of a 93-year-old female exhibited a high density of progerin-positive nuclei in the upper and lower dermis (Fig. 4B). A similar pattern of progerin distribution was detected in skin sections from different body sites (Fig. 4C) including the hand (60 years), leg (85 years) and scalp (90 years). Progerin was detected as strong nuclear rim staining in fibroblasts located in the upper, middle and deep dermal compartments in elderly individuals (Fig. 4B, and 4C), while in young individuals, a few fibroblasts accumulating progerin were localized in the papillary dermis close to the basement membrane. With increasing age, the number of positive cells increased in the upper dermis and progressively extended more deeply, creating a gradient of progerin-positive fibroblasts from the basement membrane to the reticular dermis. Skin derived from both sun-exposed areas and non-exposed areas shared a similar distribution of progerin-positive fibroblasts (Fig. 3 and 4).

### Progerin was detected in a subset of terminally differentiated keratinocytes in some skin biopsies derived from elderly individuals

**Figure 5:**
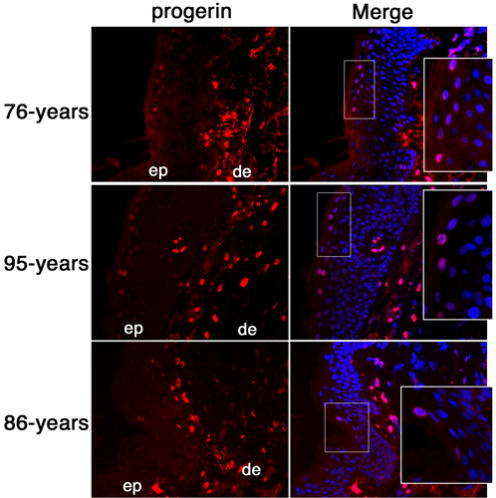
Progerin detection in a subset of terminally differentiated keratinocytes. Left panels correspond to anti-progerin monoclonal antibody staining of skin sections derived from individuals of indicated age. Right panels correspond to the merged signal of dapi and progerin signals. Square indicates the zoomed in region of the epidermis. ep denotes epidermis, and de the dermis.

In the vast majority of unaffected skin biopsies tested herein, progerin was not detected in the epidermis, as shown in Figure 3 to 4. However, in some skin biopsies derived from 70 to 95 year-old subjects, a positive progerin signal was detected within the epidermis. Representative examples of skin sections exhibiting a progerin-positive signal in the epidermis are shown in Figure 5. When progerin was detected in elderly epidermis, only a few keratinocytes per section were positively labelled with anti-progerin mAb in the uppermost layers of the epidermis and the signal was never as bright as the one harbored by dermal fibroblasts (Fig. 5). Skin sections from 76- and 95-year-old females showed the highest number of positively labelled keratinocytes in the granular layer of the epidermis (Fig. 5). However, the progerin-positive keratinocytes were not uniformly distributed throughout the entire length of the epidermis on the sections but rather were localized within a small area. In most elderly skin sections, only one to three keratinocytes were sporadically observed in the upper most layer of the epidermis, as shown on an 86 year-old male skin section (Fig. 5). In these terminally differentiated cells, progerin signal was localized into a rim-like structure at the nuclear periphery (Fig. 5). Overall, the progerin signal was weak compared to the strong signal obtained in dermal fibroblasts.

These results indicate that progerin accumulates in terminally differentiated keratinocytes that are localized within the layers closer to the skin surface. Moreover, the progerin-positive keratinocytes exhibited a thick nuclear rim staining, again reminiscent of staining observed in progerin-positive keratinocytes from HGPS skin sections (Fig. 3A). Since only very few keratinocytes from the upper epidermis were labelled with anti-progerin antibody, we could not rule out the possibility of epitope masking which is common in lamin detection. We used different fixation methods in combination with different permeabilization procedures but the staining remained unchanged (data not shown). The distribution and localization of the few progerin-positive keratinocytes were consistent with observations of skin sections derived from the patient with HGPS (Fig. 3A). As in HGPS keratinocytes, the normal keratinocytes were terminally differentiated as defined by their location in the uppermost layer of the granular layer [42]. In addition, those progerin positive-keratinocytes exhibited identical rim-like staining at the nuclear envelope periphery. While a progerin-positive signal was sporadically detected in keratinocytes from the interfollicular dermis, no signal was detected in the different epidermal appendages and vasculature system regardless of body site or age of the donor sample under our experimental conditions.

## Discussion

These data show for the first time that sporadic use of the cryptic splice site in exon 11 of *LMNA* occurs *in vivo* in normal cells, however that site is much more active in HGPS cells carrying the *LMNA* G608G mutation. Progerin mRNA transcripts were detected at very low levels in all skin samples of unaffected individuals and remained fairly constant at all ages (Fig. 1). A similar observation was previously reported using fibroblast cultures derived from healthy individuals [38].

While the progerin mRNA could be detected at uniform levels by RT-PCR analysis in all tissue samples, the protein apparently accumulated with age. Western blot analysis was capable of detecting low levels of progerin in skin samples from elderly individuals (Fig. 1C), but the protein was below limit of detection in young samples. In skin biopsies from young individuals, progerin was either absent or present only at low levels, since it was not detectable by indirect immunofluorescence (Fig. 3). With advancing age, concomitant with alterations of the dermal compartment (such as disorganized connective tissue), progerin accumulated in skin cells but was restricted to specific cell types, primarily dermal fibroblasts and terminally differentiated keratinocytes. Thus, progerin-positive cells were not randomly distributed within the skin (Fig. 3 to [Fig pone-0001269-g004] 5). In the dermis, progerin-positive fibroblasts appeared first at the basement membrane and their number increased and spread throughout the dermis with age (Fig. 3 and 4).

Dermal fibroblasts are the major cell type in the dermis and represent a heterogeneous population of cells based on differences in proliferation potential and extracellular matrix protein synthesis [43]. Our findings indicate that the population of progerin-positive fibroblasts might characterize a possible terminal differentiation phenotype of dermal fibroblasts *in vivo*. In support of this observation, our results indicate that progerin-positive cells are not randomly distributed in the dermis, but appear in an age-related fashion at the basement membrane and gradually spread throughout the dermal compartment (Fig. 3 and 4). Moreover, while a high number of progerin-positive cells were detected in skin biopsies of older individuals, only a few of them were found in the respectively derived fibroblast cultures (Fig. 2), suggesting that the progerin-positive cells might have reached terminal growth arrest, and were thus not able to propagate in culture. However, their number increased slightly in late passage cultures suggesting that progerin accumulates in cells that have entered a stage of terminal differentiation or senescence.

We and others have previously reported that progerin build-up in HGPS cells occurs in a cellular-age-dependent manner [29], [30]. Moreover, accumulation of progerin has a negative impact on cell cycle progression and elicits reduced migration in HGPS fibroblasts [30], [39]. Altogether, these findings indicate that progerin-positive fibroblasts *in vivo* might define a subpopulation of fibroblasts that have reached terminal differentiation and/or senescence.

The epidermis is a self-renewing stratified epithelium mainly composted of keratinocytes that undergo a complex and dynamic program of terminal differentiation throughout life [44]. This process starts when proliferating keratinocytes of the basal layer move upward to the suprabasal layers and progressively acquire the ability to express sequentially the specific gene products that are required for differentiation [45]. Terminally differentiated keratinocytes of the granular layer are in a transitional stage on their way to forming the cornified layer of the epidermis [44]. This transition is accompanied by the elimination of all organelles including the nuclei and changes in cytoplasmic functions. These cellular events allow the production of the outermost epidermal layer, composed of the flattened, enucleated, dead cornified cells (corneocytes) that ensure the skin barrier function [44]. Strikingly, it is within this transitional epithelial layer that progerin appears within a small subset of keratinocytes. These cells not only have reached terminal differentiation but also are about to extrude their nuclei and as such could be considered as having reached the end of their lifespan. Such terminal differentiation and transitional stages between nucleated and enucleated cells must imply not only an important rearrangement of the nucleus but must also implicate a large-scale genome reorganization designed to switch off gene expression that is no longer required. Such remodelling of the nuclear lamina composition and changes in heterochromatin epigenetic marks to silence gene expression and overall chromatin organization must synergistically and actively participate in the settings of nuclear exclusion. We suggest that during this final stage of terminal differentiation, the keratinocytes must have reached the end of their lifespan *in vivo* and may be regarded as senescent cells [46], [47].

Replicative senescence is a permanent state of proliferation arrest [48]. Terminal differentiation defines cells that permanently exit the cell cycle in the course of acquiring functional specialization. Both cellular stages share some common properties including stable cell-cycle arrest, flattened and enlarged morphology, increased cytoplasmic enzymatic vesicles, and changes in chromatin condensation and in gene expression patterns [49], [50].

Progerin is linked to the pathogenesis of HGPS and exerts a toxic nuclear effect because it remains permanently farnesylated [18]. Cells derived from subjects with HGPS exhibit dysmorphic nuclei with significant changes in nuclear shape, including nuclear envelope invaginations, thickening of the nuclear lamina, loss of peripheral heterochromatin, and clustering of the nuclear pores [29], [30]. The changes in the lamina protein composition appear to be responsible for the disruption and loss of peripheral chromatin in HGPS cells, and has been linked to impaired epigenetic histone markers and genomic instability [34].

Our findings further extend these previous studies by showing the dynamic changes in the nuclear lamina composition with the incorporation of an “age-associated” lamin isoform progerin during the process of keratinocyte differentiation *in vivo*. We postulate that, analogous to the process that occurs in HGPS cells, the build-up of progerin in the nuclear lamina in normal cells contributes to the delocalisation of heterochromatin clusters away from the nuclear periphery, thereby contributing to the large scale-genome decondensation to allow genomic reorganization. This reorganization is requisite to ensure terminal differentiation and/or senescence. Finally, the association between progerin build-up to terminal differentiation and/or senescence in normal cells suggests that in the context of HGPS cells, progerin accumulation could trigger abnormal differentiation and early senescence which would prematurely deplete the pool of mitotic cells with renewal potency from tissue and cause the clinical sequelae that characterize old age.

As progerin appears to be a cellular aging biomarker for dermal fibroblasts and keratinocytes of normal individuals *in vivo*, we are drawn to the conclusion that HGPS may indeed be a useful model in which to learn more about normal aging. Therapeutic strategies already tested on HGPS cells, such as farnesyltransferase inhibitors, might even be useful in preventing normal aging and possibly slowing progression of other age-related pathologies.

## Materials and Methods

### Production and characterization of a rabbit monoclonal anti-lamin A G608G antibody

The lamin A G608G amino acid sequence reading frame was determined previously [27], [51]. To generate a specific anti-Lamin A G608G antibody, we chose a short peptide overlapping the region where the 50 amino acid internal deletion occurred in lamin A mutant G608G sequence as described previously [30]. Three rabbits were immunized with the peptide using the standard protocol performed by Covance ImmunoTechnologies (Denver, PA, USA). Preimmune and immune sera were characterized by Western blot analysis and indirect immunofluorescence on HGPS and control fibroblast cells. The serum of rabbit 972 specifically recognized progerin protein and gave no signal with A-type lamin or pre-lamin A. The spleen from Covance rabbit 972 was sent to Epitomics, Inc (Burlingame, CA, USA). Lymphocytes were isolated from the spleen and fusion was performed according to Epitomics' standard protocol. Positive hybridomas were selected and the supernatants of the primary clones and subsequent subclones were screened by Western blot and indirect immunofluorescence analysis using control and HGPS dermal fibroblasts treated or untreated with FTI as described previously [30]. One clone, 972S9, was selected based on its specific reaction for progerin and was used in this study.

### Human skin biopsy sections and primary dermal fibroblast cultures

Normal skin biopsies and newborn foreskins were obtained from the Dermatology Clinic in accordance with the health research ethics board of Columbia University. The biopsies originated from different body sites and were obtained from equal numbers of males and females. The sex, age and body site of each donor was recorded (Table 1). Skin biopsies were embedded in Optimum Cooling Temperature medium (O. C. T.) and cryopreserved for tissue sectioning. Serial 6 µm frozen skin sections were prepared and stored at −80°C. In addition, small pieces were snap frozen in liquid nitrogen at the time of their collection for mRNA and protein extractions. Primary cultures of dermal fibroblasts were established using explant culture or enzymatic digestion of the skin as described previously [41].

Briefly, skin tissue was rinsed in PBS and incubated overnight in a Dispase II solution at 4°C. The epidermis and dermis were mechanically separated and primary HDF cultures were established using explants or enzymatic digestion methods of the dermis. For explant culture, the dermal portion of the skin was washed in PBS supplemented with penicillin and streptomycin cut into small pieces, and spread onto a culture dish. Culture medium, DMEM supplemented with 15% fetal bovine serum, was added. Fibroblast outgrowth started at day 3 to 7; the skin pieces were removed after a week and cultures were grown to 70% confluence. For HDF cultures established by enzymatic dissociation of the skin, dermal pieces were transferred to a solution of Collagenase I (Worthington Biochemical) at 200 units/mL of 1× PBS including 0.3 mM CaCl_2_. The pieces were kept at 37°C shaking for 1 to 4 hours until they were digested. The solution with the dermal fragments was then diluted 5-fold with complete DMEM medium (15% FBS, 1% penicillin-streptomycin, 1% L-glutamine), passed over a 70 µm cell strainer (BD Falcon) and centrifuged. The cell pellet was resuspended in complete medium and the cells were plated, grown to 70–80% confluence and cryopreserved at −80°C.

### HGPS skin biopsy and dermal fibroblast cells

Dermal fibroblasts derived from HGPS patients carrying the *LMNA* mutation G608G (HGADFN001, HGADFN003, and HGADFN127) were grown as described previously [30]. The Progeria Research Foundation kindly provided the HGPS cells and frozen skin sections derived from a skin biopsy of a 9 year-old donor with HGPS carrying *LMNA* G608G mutation (HGADFN143).

### One step-Reverse Transcription/Polymerase Chain Reaction (RT-PCR)

Total RNA was extracted from skin biopsy pieces using an RNA extraction kit as per the manufacturer (QIAGEN). 1 µg of RNA from each sample was submitted to one step RT-PCR (OneStep RT-PCR, QIAGEN) using primers located in exon 9 and 12 of lamin A (forward primer: 5′ GGCTGCGGGAACAGC 3′, and reverse primer: 5′ CTGGCAGGTCCC 3′) described previously [38], together with primers located in human β actin isoform (Forward: CCCAGCACAATGAAGATCAA and reverse GTGTAACGCAACTAAGTCAT) as internal control. 50 µl reactions were submitted to reverse transcriptase for 30 minutes at 50°C, followed by PCR activation step at 95°C for 15 minutes. PCR conditions were 35 cycles, each cycle consisting of 30-sec denaturation at 94°C, 30-sec annealing at 55°C, and 30-sec polymerization at 72°C. 15 µl of the reaction was analysed on 2% agarose gel and stained with ethidium bromide.

### Indirect immunofluorescence

Primary cultures of dermal fibroblasts from patients and controls were processed for indirect immunofluorescence as described previously [51]. Mouse anti-lamin A Jol4 (Serotec) or clone 133A2 (abcam), anti-lamin A/C 131C3 (abcam), and anti-human α smooth muscle actin, clone 1A4 (DakoCytomation) were purchased. The secondary antibodies were affinity purified Alexa Fluor 488 goat or donkey IgG antibodies (Molecular Probes) and Cy3-conjugated IgG antibodies (Jackson ImmunoResearch laboratories). All samples were also counterstained with DAPI (Sigma-Aldrich).

Immunohistochemistry was performed on 6 µm frozen sections fixed by methanol/acetone (1V/1V) at −20°C for 10 minutes and washed in PBS, then blocked in PBS buffer containing 3% BSA, 10% normal goat serum and 0.3% Triton X-100 for 30 minutes and 1 hour in the same buffer without Triton X-100. Slides were incubated with the monoclonal anti-progerin antibody for 1 hour. After 6 washes in blocking buffer, slides were incubated with donkey anti-rabbit affinity purified Cy3-conjugated IgG antibodies. Slides were washed in blocking buffer and in PBS, then mounted with Vectashield mounting medium (Vector Inc.)

### Western blot analysis

Skin tissues were extracted in cytoskeleton buffer (CSK) (100 mM NaCl, 300 mM sucrose, 10 mM PIPES (pH 6.8), 3 mM MgCl_2_, 0.5% triton X-100) and protease inhibitors (Roche) for 15 minutes on ice. Skin pieces were minced and homogenized with PowerGen 125 (Fisher Scientific) in CSK buffer. The insoluble material was digested in PBS buffer containing 100 µg/ml of DNAse I and 100 µg/ml of RNAse A (Sigma) for 20 minutes at 20°C; after centrifugation the remaining pellet was resuspended in Laemmli sample buffer (BioRad) and boiled 5 minutes at 95°C. Equal amounts of extracts were loaded in parallel on a 7.5% polyacrylamide gel. After separation by electrophoresis, proteins were transferred to nitrocellulose membranes and incubated with blocking buffer as described previously [30]. Membranes were incubated with primary antibodies (Ab 972S9, anti-lamin A/C kindly provided by Dr. N. Chaudhary, anti-actin Ab (Sigma)), washed, and then incubated with the corresponding secondary antibody coupled to horseradish peroxidase (Jackson ImmunoResearch Laboratories). Proteins were visualized using the enhanced chemiluminescence detection system (Amersham Pharmacia Biotech).

Western blot analysis of progerin expression in normal fibroblast cultures established from an 86-year-old female was performed after immunoprecipitation of nuclei extract prepared from early (PPD 15) and late population doublings (between 30 to 35 PPDs). Nuclei were isolated from normal cell pellets, containing an average of 6×10^6^ cells, and from HGPS cell pellets, containing 2×10^6^ cells at early PPDs (below 25), in buffer A (10 mM HEPES (pH7.9), 1.5 mM MgCl_2_, 10 mM KCL) supplemented with protease inhibitor cocktail tablet (Roche Applied Science). After 10 minutes incubation on ice, the preparation was dounce homogenized by 25 strokes followed by 10 minutes centrifugation at 800g at 4°C. Nuclei pellets were resuspended in RIPA buffer supplemented with protease inhibitors and were briefly sonicated on ice and centrifuged at 4°C. Extracts were incubated with 10 µg of purified 972S9 IgG (anti-progerin rabbit monoclonal) for 4 hours at 4°C; 30 µl of protein A sepharose, equilibrated in RIPA buffer, was added and the extracts were incubated for another hour at 4°C. After four washes in RIPA buffer and two washes in PBS, the IP pellets were resuspended in 50 µl Laemmli sample buffer (BioRad), separated on SDS-PAGE gels, and transferred onto nitrocellulose. Westerns were probed with anti-progerin rabbit mAb 972S9 and further processed as described above.
